# In Silico Analysis of Ion Channels and Their Correlation with Epithelial to Mesenchymal Transition in Breast Cancer

**DOI:** 10.3390/cancers14061444

**Published:** 2022-03-11

**Authors:** K. T. Shreya Parthasarathi, Susmita Mandal, Smrita Singh, Seetaramanjaneyulu Gundimeda, Mohit Kumar Jolly, Akhilesh Pandey, Jyoti Sharma

**Affiliations:** 1 Institute of Bioinformatics, International Technology Park, Bangalore 560066, India; shreya@ibioinformatics.org (K.T.S.P.); smrita@ibioinformatics.org (S.S.); seetaram@ibioinformatics.org (S.G.); 2 Center for BioSystems Science and Engineering, Indian Institute of Science, Bangalore 560012, India; susmitam@iisc.ac.in (S.M.); mkjolly@iisc.ac.in (M.K.J.); 3 Department of Laboratory Medicine and Pathology, Mayo Clinic, Rochester, MN 55905, USA; pandey.akhilesh@mayo.edu; 4 Center for Molecular Medicine, National Institute of Mental Health and Neurosciences (NIMHANS), Hosur Road, Bangalore 560029, India; 5 Manipal Academy of Higher Education (MAHE), Manipal 576104, India; 6 Center for Individualized Medicine, Mayo Clinic, Rochester, MN 55905, USA

**Keywords:** RNA-Seq, microarray, membrane proteins, bioinformatics, interaction networks, prognosis

## Abstract

**Simple Summary:**

Breast cancer involves changes in the healthy cells of the breast resulting in rapid and abnormal division of cells that later spread to other parts of the body through the process of metastasis, which involves epithelial mesenchymal transition (EMT). Ion channels play a significant role in the switch from epithelial to mesenchymal transition through their contributions to cellular motility, cell volume regulation and cell cycle progression. Comprehensive computational analyses were performed to understand the role of ion channels in tumor/metastatic samples of breast cancer and their correlation with EMT.

**Abstract:**

Uncontrolled growth of breast cells due to altered gene expression is a key feature of breast cancer. Alterations in the expression of ion channels lead to variations in cellular activities, thus contributing to attributes of cancer hallmarks. Changes in the expression levels of ion channels were observed as a consequence of EMT. Additionally, ion channels were reported in the activation of EMT and maintenance of a mesenchymal phenotype. Here, to identify altered ion channels in breast cancer patients, differential gene expression and weighted gene co-expression network analyses were performed using transcriptomic data. Protein–protein interactions network analysis was carried out to determine the ion channels interacting with hub EMT-related genes in breast cancer. Thirty-two ion channels were found interacting with twenty-six hub EMT-related genes. The identified ion channels were further correlated with EMT scores, indicating mesenchymal phenotype. Further, the pathway map was generated to represent a snapshot of deregulated cellular processes by altered ion channels and EMT-related genes. Kaplan–Meier five-year survival analysis and Cox regressions indicated the expression of *CACNA1B*, *ANO6*, *TRPV3*, *VDAC1* and *VDAC2* to be potentially associated with poor survival. Deregulated ion channels correlate with EMT-related genes and have a crucial role in breast cancer-associated tumorigenesis. Most likely, they are potential candidates for the determination of prognosis in patients with breast cancer.

## 1. Introduction

Breast cancer is a life-threatening disease and is one of the most common types of cancer prevalent in individuals of all age groups, with the majority of cases in females. It is also one of the most aggressive tumors with multifaceted gene expression levels at different stages of tumor progression. Though breast cancer starts as a local disease, mutations occur at distinct junctures of tumor differentiation, facilitating tumor cells to metastasize [1]. The process of metastasis starts locally by invading the host tissues surrounding the primary tumor and into the blood or lymphatic vessels through a mechanism known as epithelial-mesenchymal transition (EMT) [2].

Ion channels are multimeric proteins located in the plasma membrane of the cell. They form a passageway extending from one side of the membrane to the other, thereby allowing the flow of ions into and out of the cell based on the electrochemical gradient. This generates a membrane potential that mediates a large number of biological functions within cells and across cell membranes [3]. Recent evidence has shown that ion channels are involved in the progression and pathology of various cancers [4,5,6]. *Eag1* (*KCNH1*), a potassium channel, was reported to be involved in the proliferation and cell cycle of liposarcoma cells [7]. *TRPM1*, a cation-permeable channel, was found to be a prognostic marker for metastasis in melanoma [8]. *TRPV6* was implicated in prostate adenocarcinoma and colorectal cancer cell lines [8]. Additionally, studies carried out by Ko et al. reported the association of several ion channels with various pathological features in breast cancer [3]. The activation of EMT programs is an expository mechanism for the gain of malignant phenotypes by epithelial cancer cells [9]. Often, this activation relies on signaling events between cancer cells and neighboring stromal cells [10]. Specific ion channels in various aspects of EMT induction have been reported, including the downregulation of *CFTR*, an ion channel that promoted EMT, migration and invasion in breast cancer [11]. Knockdown of hERG1 (*KCNH2*) expression led to reversion of the EMT profile in colorectal cancer cell lines, leading to reacquisition of the epithelial-like profile [12]. Expression of EMT transcription factors together with overexpression of *TRPV1* cation channels led to hepatocarcinogenesis. Further inhibition of *TRPV1* inhibited the growth of hepatocellular carcinoma cells [13]. The upregulation of *ASIC1* and *ASIC3* led to acidity-induced EMT through elevation of intracellular Ca^2+^ concentration in pancreatic cancer cells. It was also shown that *ASIC1* and *ASIC3* positively correlated with mesenchymal marker vimentin and inversely correlated with epithelial marker E-cadherin in those cells [14].

The current study aims to identify altered ion channels and ion channels co-expressed with EMT-related genes in patients with breast cancer. Several bioinformatic analysis of ion channels and EMT-related genes led to the identification of altered ion channels in tumor/metastatic samples along with ion channels co-expressed with EMT-related genes in breast cancer patients. EMT scoring is a promising and versatile tool for the systematic investigation of EMT dynamics in cancer progression [15]. EMT scores for the expression profiles were calculated using 76-gene EMT signature based (GS76), multinomial logistic regression-based (MLR) and Kolmogorov–Smirnov test-based (KS) metrics [16]. Protein–protein interactions (PPIs) network analysis revealed 32 ion channels that interacted with 26 hub EMT-related genes in breast cancer. The correlation of potential ion channels with the EMT scores was used to statistically evaluate their potential in the EMT program. Furthermore, data mining of identified ion channels revealed the possible repercussions of altered expression of ion channels in cellular processes. A pathway map depicting various reactions, including the role of ion channels in serotonin signaling, insulin signaling, calcium signaling, adipocyte metabolism, nitric oxide signaling, glutamatergic signaling and osmotic stress, was generated. *CACNA1B*, *ANO6*, *TRPV3*, *VDAC1* and *VDAC2* were found to be prognostically significant. These analyses could thus identify ion channels that can be further studied to validate their potential role as molecular markers of breast cancer. Targeting these ion channels might lead to suppression of tumor growth. Understanding the role of these ion channels and their detailed mechanism with EMT programs may pave the way for developing new therapeutic strategies to improve the clinical outcomes of patients with breast cancer. 

## 2. Material and Methods

**Figure 1:**
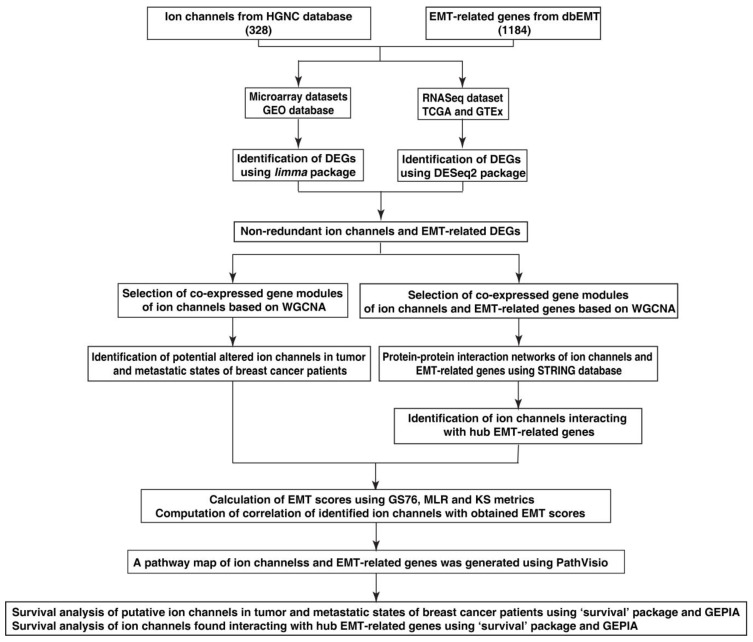
Workflow for the identification of potential ion channels and its interaction with EMT-related genes in tumor and metastatic samples of patients with breast cancer.

The workflow of this study is depicted in Figure 1.

### 2.1. Data Collection

Primarily, a list of 328 ion channels was taken from the HGNC database [17] and 1184 EMT-related genes from dbEMT (version 2.0) [18]. Publicly available transcriptomic datasets were analyzed to investigate gene expression in breast cancer. RNA sequencing data (RNA-Seq) of breast cancer patients and normal individuals corresponding to the ion channels and EMT-related genes were downloaded from the UCSC Xena portal, including the Cancer Genome Atlas (TCGA) and Genotype Tissue Expression (GTEx) data [19]. The number of metastatic samples obtained in the RNA-Seq dataset was limited; thus, the inclusion of microarray samples was taken into consideration. Microarray datasets GSE42568 and GSE52604 were retrieved from the Gene Expression Omnibus (GEO) database. The detailed description regarding the datasets have been mentioned in Tables S1–S3.

### 2.2. Data Pre-Processing and Identification of Differentially Expressed Genes

The log (read count + 1) normalized RNA-Seq data were transformed to raw reads for input in DESeq2 [20]. The probe IDs of microarray datasets were converted to gene symbols using the annotation files GPL570 ((HG-U133_Plus_2) Affymetrix Human Genome U133 Plus 2.0 Array) and GPL6480 (Agilent-014850 Whole Human Genome Microarray 4x44K G4112F) obtained from the GEO database. Gene symbols were subsequently superimposed with the list of ion channels and EMT-related genes. Microarray expression profiles corresponding to those genes were set apart for the detection of differentially expressed genes (DEGs). DEGs for ion channel and EMT gene sets were identified individually. R Bioconductor package DESeq2 was used for RNA-Seq data, and the *limma* (v3.28.14) package [21] was used for the identification of DEGs in microarray datasets. In RNA-Seq and GSE42568 datasets, DEGs were identified for 3 subgroups—Normal vs. Tumor (HT), Tumor vs. Metastatic (TM) and Normal vs. Metastatic (HM). GSE52604 contained only metastatic and normal samples. The genes with an adjusted *p*-value (*p*adj) ≤ 0.05 and log_2_ fold change (log_2_FC) > 0.6 were selected as upregulated and the genes with a *p*adj ≤ 0.05 and log_2_FC < −0.6 were selected as downregulated genes. A non-redundant list consisting of all DEGs for the three subgroups was prepared (Tables S4 and S5).

### 2.3. Construction of Co-Expression Networks of Altered Ion Channels and EMT-Related Genes

The expression profiles of the DEGs were further employed for the construction of scale-free co-expression networks using the weighted gene co-expression network analysis (WGCNA) R package (v4.0.0) [22,23]. Since the datasets belonged to different platforms, they were analyzed independent of each other. First, co-expression networks for DEGs in ion channels were constructed on the three datasets. Later, a combined gene set comprising both ion channels and EMT-related genes was formed for co-expression network construction. The genes in each of the datasets were filtered using goodSamplesGenes function in R. The adjacency correlation matrix was calculated based on the scale-free network model [23]. A suitable soft threshold power was selected as the soft-thresholding parameter to ensure a scale-free network using the pickSoftThreshold function. The obtained adjacency matrix was further used to derive the signed Topological Overlap Matrix (TOM) and the corresponding dissimilarity matrix (1-TOM). Based on the dissimilarity matrix, hierarchical clustering was performed to group genes with similar expression profiles into the same gene modules using the DynamicTreeCut algorithm [24].

For the selection of candidate modules, expression profiles of each module were summarized by module eigengenes (MEs) and correlated with the binary trait (normal, tumor, metastatic) values. Thus, the module-trait relationship was obtained, and the *p*-value was calculated as a confidence measure. The association of individual genes in the module with the binary trait was quantified by the Gene Significance (GS) value. Module membership (MM) was calculated as the correlation of gene expression profiles with the MEs. Further, intramodular gene connectivity with the binary trait was established using GS vs. MM plots [23]. Finally, the best correlated modules with a significant *p*-value were selected from each subgroup in each dataset. Parameters adjusted at each step of WGCNA are provided in Tables S7 and S8. The obtained networks of combined gene sets were exported to Cytoscape with the weight threshold value set to 0.02 for visualization.

### 2.4. Identification of Altered Ion Channels in Tumor and Metastatic States

The modules chosen subsequent to WGCNA of ion channels were examined for the identification of ion channels in tumor and metastatic states. The overlapping ion channels in the selected modules of three datasets belonging to HT were shortlisted as ion channels involved in the process of transition from normal to tumor. Similarly, overlapping ion channels in TM were selected as ion channels in the transition between the tumor to metastatic phenotype of breast cancer.

### 2.5. EMT Score Calculation

EMT scores of the transcriptomic profiles of samples from patients with breast cancer were calculated using previously developed methods, GS76, MLR and KS [16,25,26]. A well-defined set of gene signatures along with a classification algorithm forms the basis for EMT score calculation in these methods. A higher GS76 score indicates a more epithelial sample, but a higher MLR and KS score indicates a more mesenchymal sample. MLR quantifies the extent of EMT on a scale of [0, 2], and KS scores are defined on a scale of [−1, +1] [16].

EMT scoring methods aid in quantifying the extent of EMT phenotype of a sample. Different methods based on different gene signatures and algorithms have been developed for this purpose, due to which the scores obtained from these methods may vary for the same dataset. However, computing the scores with more than one method may capture the extent of EMT potential in all the genes involved in EMT process, thus expanding the search space for further analysis.

### 2.6. Protein–Protein Interaction Networks of Altered Ion Channels Co-Expressed with EMT-Related Genes

Protein–protein interaction networks (PPINs) of the selected modules obtained through WGCNA of the combined gene set were constructed using the STRING (v11.0) database [27] to deduce the association through known and predicted interactions between the genes in the modules. The interactions with a medium confidence and FDR of 5% were visualized in Cytoscape (v3.8) [28] and Gephi (v0.9.2) software. To further identify ion channels interacting with hub EMT-related genes, the networks were analyzed using NetworkAnalyser [29], a Cytoscape plugin that calculates the various properties of a network, including degree centrality, betweenness centrality and closeness centrality. The top 15 genes with the highest degree centrality measure were selected as hub genes from each module. Thereafter, the ion channels interacting with these genes were shortlisted.

### 2.7. Computation of Correlation of EMT Scores with Identified Ion Channels

The EMT scores of each sample estimated by the GS76, MLR and KS methods (mentioned in Section 2.5) were correlated with the expression profiles of the potential ion channels interacting with EMT-related genes and in tumor/metastatic states of breast cancer. Correlating EMT scores with expression profiles of genes of interest provides statistical evidence that supports the selection of those genes for further assessment.

### 2.8. Generation of Pathway Map of Altered Ion Channels and EMT-Related Genes

A data mining approach was used to annotate reactions involving identified ion channels and EMT-related genes in various cellular processes in patients with breast cancer. The articles were searched and screened from the PubMed database pertaining to possible effects of alterations in ion channels and EMT-related genes in breast cancer patients. Furthermore, various reactions, such as activation, inhibition and transportation, were annotated [30,31,32,33]. These reactions describe the translocation of ions and small molecules between subcellular compartments through ion channels and the role of ion channels with EMT-related genes in several cellular processes. Thereafter, a pathway map depicting the annotated reactions involving identified ion channels and EMT-related genes was generated in Graphical Pathway Markup Language (GPML) format using PathVisio (version 3.3.0) [34]—an opensource pathway drawing software. Nodes describe the entities (proteins, genes) and edges represent the relationship between the nodes in the map.

### 2.9. Survival Analysis of Identified Altered Ion Channels

The R Bioconductor “RTCGA” package was utilized to obtain the clinical data for survival analysis. Cox proportional hazard regression analysis was performed to determine the correlation between gene expression and 5-year survival rate of patients with breast cancer. The “survival” R Bioconductor package was used to calculate the log rank *p*-values and the hazard ratios (HR) with a confidence interval of 95%. The survival differences between high and low expressions of the putative ion channels were visualized by generating Kaplan–Meier (KM) survival plots using the R Bioconductor “survminer” package. The group cut-off criteria were set to median value. Further, the relationship between overall survival (OS) and expression profiles of putative ion channels was determined by KM OS plots using Gene Expression Profiling Interactive Analysis (GEPIA) [35]. GEPIA is a web server for interactive analysis of cancer and normal gene expression profiles. It analyses the dependency of OS of patients on high and low expression of genes. The calculation of HR was set to the Cox PH model with a confidence interval of 95%, and group cut-off criteria were again set to the median value.

## 3. Results

### 3.1. Differentially Expressed Ion Channels and EMT-Related Genes

**Figure 2:**
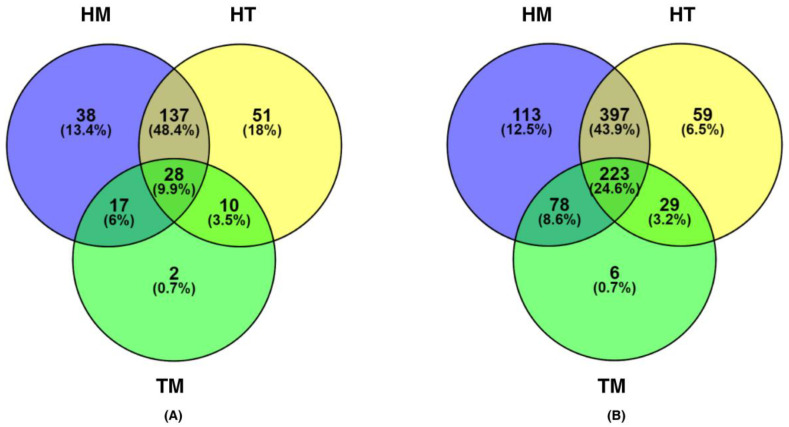
Overlap of differentially expressed genes among HM, HT and TM. (**A**) Venn diagram depicting total number of differentially expressed ion channels in the RNA-Seq and two microarray datasets (GSE42568 and GSE52604) in HM (blue), HT (yellow) and TM (green). (**B**) Venn diagram showing the total number of differentially expressed EMT-related genes in the RNA-Seq and two microarray (GSE42568 and GSE52604) datasets in HM (blue), HT (yellow) and TM (green).

DESeq2 analysis of the RNA-Seq dataset with ion channels resulted in 225, 57 and 141 DEGs and the dataset with EMT-related genes resulted in 607, 336 and 601 DEGs in HT, TM and HM, respectively. The number of ion channels found to be differentially expressed in the microarray dataset (GSE42568) was 28 and 29 in HT and HM in analysis using the *limma* package (v3.28.14). In HT and HM of GSE42568, 326 and 344 differentially expressed EMT-related genes were obtained. Similarly, 155 ion channels and 470 EMT-related genes were found in HM of microarray dataset (GSE52604) (Table 1). In subgroups HT, TM and HM, 226, 57 and 220 ion channels, respectively, were obtained as non-redundant DEGs. Likewise, 708, 336 and 811 EMT-related genes were obtained (Figure 2). Ten genes were identified as common DEGs in both ion channel and EMT lists (Supplementary Table S6). The common DEGs included aquaporins—*AQP3*, *AQP5*, *AQP9*, gap junctions—*GJB1*, *GJB2*, potassium channels—*KCNN4*, *KCNH1*, cation channels—*TRPC5*, *TRPM8*, and other channels—*CFTR*, *GRIN1*.

### 3.2. Altered Ion Channels in Tumor and Metastatic States of Breast Cancer

Identification of altered ion channel modules through WGCNA analysis of the differentially expressed ion channels and further selection of ion channels based on the obtained overlaps among the datasets led to the identification of altered ion channels in tumor and metastatic states of breast cancer.

#### 3.2.1. Identification of Altered Ion Channel Modules by Weighted Gene Co-Expression Network Analysis

**Figure 3:**
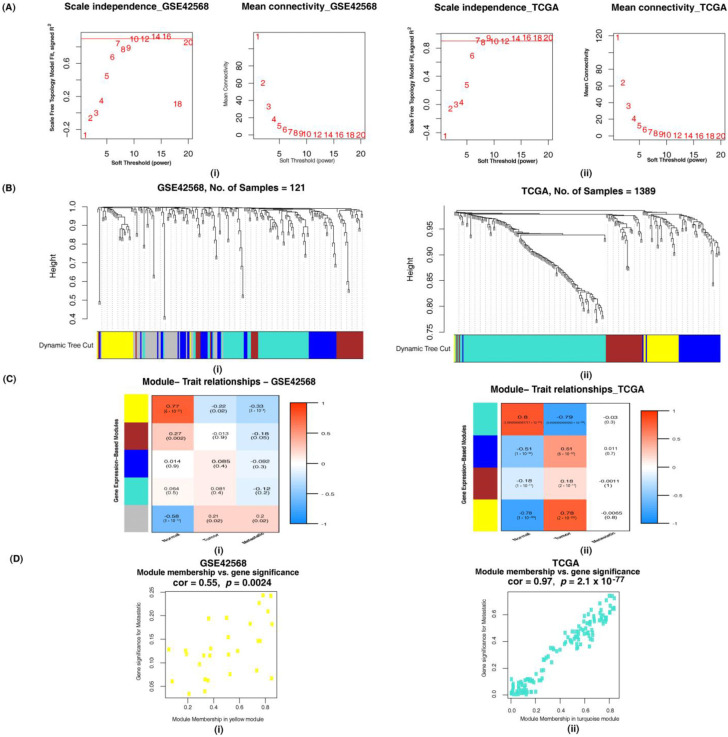
Co-expressed ion channel modules based on the non-redundant HT DEGs. (**A**) Soft-thresholding power to ensure scale-free network model: (**i**) A soft-thresholding power of 10 was chosen in the microarray dataset (GSE42568) corresponding to non-redundant HT DEGs; (**ii**) A soft-thresholding power of 7 was chosen in the RNA-Seq dataset corresponding to non-redundant HT DEGs (**B**) Hierarchical clustering of genes into modules. Modules are assigned different colors as depicted in the horizontal bar below the tree diagram: (**i**) 4 modules (yellow, blue, turquoise and brown) were obtained in the microarray dataset (GSE42568); (**ii**) 4 modules (turquoise, brown, yellow and blue) were obtained in RNA-Seq. (**C**) Correlation between module eigengenes and binary traits—normal, tumor and metastatic. Rows correspond to modules depicted as different colors and columns are the binary traits. Numbers in each cell are the correlation coefficient between module eigengenes and the binary traits and the corresponding *p*-value. (**i**) The yellow module was chosen as a significant module from the microarray dataset (GSE42568). (**ii**) The turquoise module was chosen as a significant module from RNA-Seq. (**D**) Scatter plot of gene significance (GS) for the binary trait vs. the module membership (MM) in the selected module. (**i**) GS-MM plot of the yellow module in the microarray (GSE42568) dataset. (**ii**) GS-MM plot of the turquoise module in the RNA-Seq dataset.

The soft-thresholding powers chosen to ensure a scale-free network model and the total number of modules obtained using the DynamicTreeCut algorithm of the hierarchical clustering method are provided in Table S7. The heatmap revealed the association of each module with normal, tumor and metastatic phenotypes along with *p*-values as confidence measures. Assessment of the modules using GS vs. MM plots revealed the intramodular gene connectivity with respect to a particular binary trait. Based on these criteria, selecting one module, each having a better co-relation and *p*-value resulted in 8 modules for further analysis (Figure 3 and Figure S1).

#### 3.2.2. Overlapping Ion Channels in GSE42568, GSE52604 and TCGA Datasets

Twenty-two ion channels overlapped in the selected modules corresponding to the three datasets in the HT subgroup (Table 2). From the 22 HT ion channels, 14 were reported previously as significant in tumor initiation and growth in various tumor types. Among those, four ion channels were studied in breast cancer tumor growth. Another set of 22 ion channels were similarly identified in the TM subgroup (Table 3). Of these, 15 were reported to be involved in metastasis in various cancers. This also included 6 ion channels that were reported to play a role in breast cancer progression and metastasis.

### 3.3. Identification of Altered Ion Channels Interacting with EMT-Related Genes

WGCNA of DEGs obtained from the combined gene set dataset and further PPIN analysis of significantly co-expressed modules led to the identification of altered ion channels interacting with EMT-related genes.

#### 3.3.1. Weighted Gene Co-Expression Network Analysis for the Identification of Altered Combined Gene Set Modules

The soft-thresholding powers chosen, the number of modules obtained and the selected significant combined gene set modules are provided in Table S8 and Figure S2. On the basis of the module-trait relationship and GS vs. MM plots, 8 modules with a better co-relation and *p*-value were chosen for further analysis.

#### 3.3.2. Network Analysis of Altered Ion Channels Co-Expressed with EMT-Related Genes

The modules obtained upon performing WGCNA for the combined gene set represent groups of highly co-expressed genes, although a large number of ion channels were found to be differentially expressed and co-expressed in individual analyses of the expression profiles. Only a subset of ion channels clustered together with EMT-related genes when co-expression analysis of the combined gene set was performed (Figure S3). The altered ion channels clustered with EMT-related genes were the ion channels that were co-expressed with EMT-related genes and could be involved in the EMT process. The role of TRPM7 in EMT through associations with EGF reported by Davis et al. or the induction of EMT by TGF-β involving CFTR reported by Zhang et al. expounds such notable contributions of ion channels in the EMT program [36,37].

**Figure 4:**
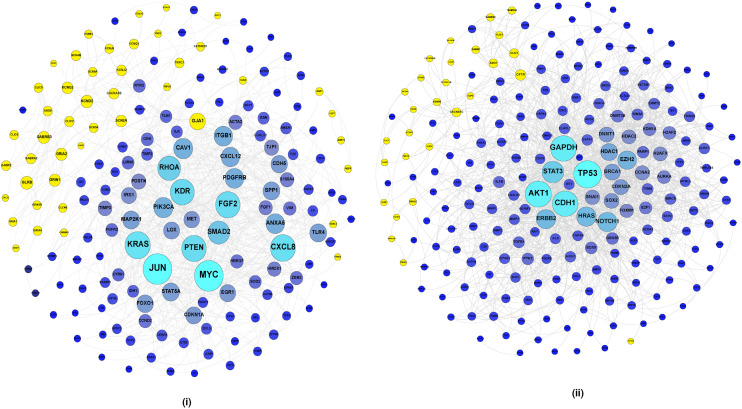
Representation of protein–protein interaction networks (PPINs) of combined gene set. Nodes in the shade of blue represent EMT-related genes and yellow nodes represent ion channels. The nodes are arranged based on the degree centrality measure. Larger nodes represent nodes with high degree centrality. (**A**) PPINs for microarray dataset (GSE42568) based on the list of non-redundant HT DEGs. (**B**) PPINs for microarray dataset (GSE42568) based on the list of non-redundant HM DEGs.

PPI networks through STRING analysis of the combined gene set selected modules revealed the predicted interactions between the genes in the module (Figure 4 and Figure S4).

Further, the PPINs of combined gene set analyzed using NetworkAnalyser resulted in the identification of hub genes in the network. Hub genes in a co-expression network are the highly interconnected nodes in a module. Out of the top 15 selected hub genes in HT of the microarray dataset (GSE42568), ten EMT-related genes were found to interact with ion channels. Of these, nine EMT-related genes were connected to *GJA1* (Table 4). Eight genes from HT of RNA-Seq (TCGA) were found to interact with ion channels (Table 5). Similarly, in HM of GSE42568, eight genes were linked to ion channels and *CFTR* was connected to four of them (Table 6). HM of the microarray (GSE52604) showed only five genes that interacted with ion channels and RNA-Seq (TCGA) showed seven genes as connected to ion channels (Table 7 and Table 8). Six ion channels interacting with EMT-related genes were common to ion channel identified in normal to tumor state of breast cancer and two ion channels were common to the ion channels identified in tumor to metastatic state of breast cancer. Thus, overall, 32 ion channels were found interacting with 26 hub EMT-related genes.

Several ion channels altered in breast cancer patients were found to interact with multiple EMT-related genes. GJA1 and GJB2 belong to the gap junction family. GJA1 was found to interact with the hub EMT-related genes JUN, MYC, FGF2, PTEN, KDR, RHOA, CAV1, ITGB1 and CXCL12. GJB2 was found in interactions with AKT1 and CDH1. TRPC6 and TRPC1 belong to the family of transient receptor cation channels and control the flow of calcium ions. TRPC6 was found to be interacting with JUN and RHOA and TRPC1 was found to be connected to RHOA and CAV1. VDAC1 is a voltage-dependent anion channel and was linked to GAPDH, HSP90AA1 and HSPA4. KCNH2, a voltage-gated potassium channel, was found to be linked with SRC, HSPA4 and HSP90AA1. CFTR is one of the most widely studied ion channels, dysregulation of which has been reported in various pathophysiological conditions [37,38]. It interacted with TP53, GAPDH, BRCA1 and DNMT1. ANO1, a calcium-activated chloride channel, was connected to CDH1 and ERBB2. AQP5 is an aquaporin involved in the movement of water across cell membranes [39]. It interacted with GAPDH and CDH1. CLIC1, a chloride channel, was found interacting with AKT1 and GAPDH.

### 3.4. Correlation of Identified Ion Channels with EMT Scores

**Figure 5:**
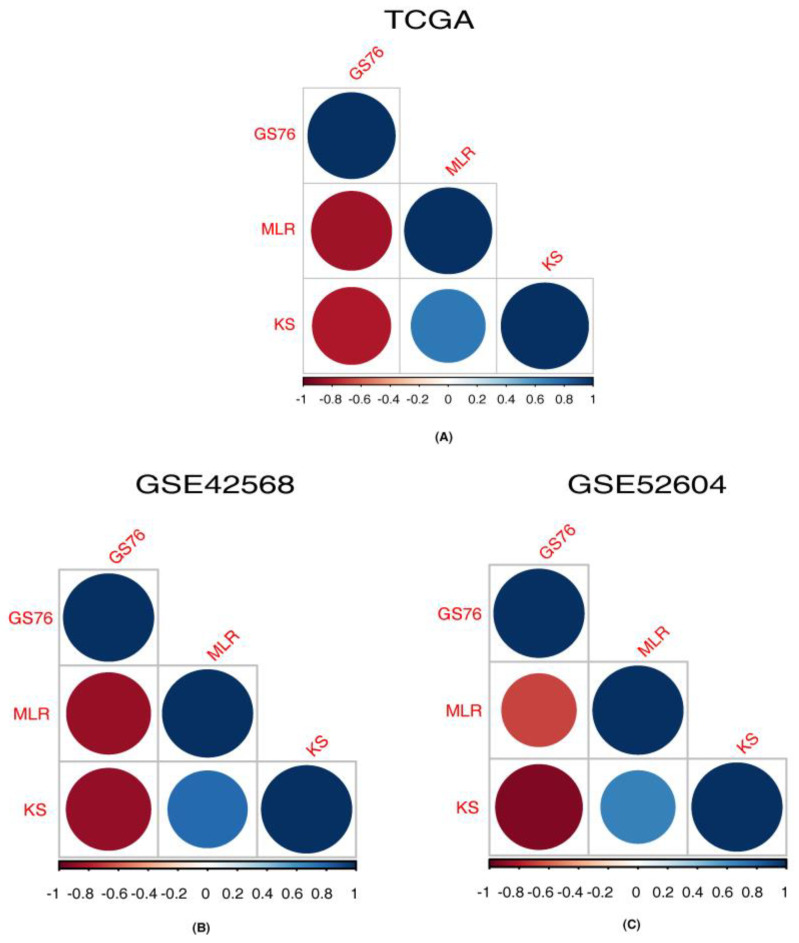
Correlation between GS76, MLR and KS scoring methods for samples from different platforms. (**A**) Correlation between GS76, MLR and KS methods for samples from RNA-Seq TCGA data. (**B**) Correlation between GS76, MLR and KS methods for samples from GSE42568 microarray data. (**C**) Correlation between GS76, MLR and KS methods for samples from GSE52604 microarray data.

The EMT scores obtained using GS76, MLR and KS methods for each sample in RNA-Seq and microarray datasets are provided in Tables S9–S11. The samples with a negative GS76 score, a positive KS score and a higher MLR score can be interpreted as mesenchymal samples. Subsequently, the samples with a positive GS76 score, a negative KS score and a lower MLR score could be an epithelial sample. The correlation between the scores relative to the specific method in each dataset was calculated and is depicted in Figure 5 and provided in Table 9. KS and MLR scores correlated positively with each other and GS76 scores correlated negatively with both KS and MLR scores.

Further, the correlations between the obtained EMT scores from each method and the expression levels of the identified ion channels in tumor, metastatic and ion channels interacting with EMT-related genes were computed (Table S12).

**Figure 6:**
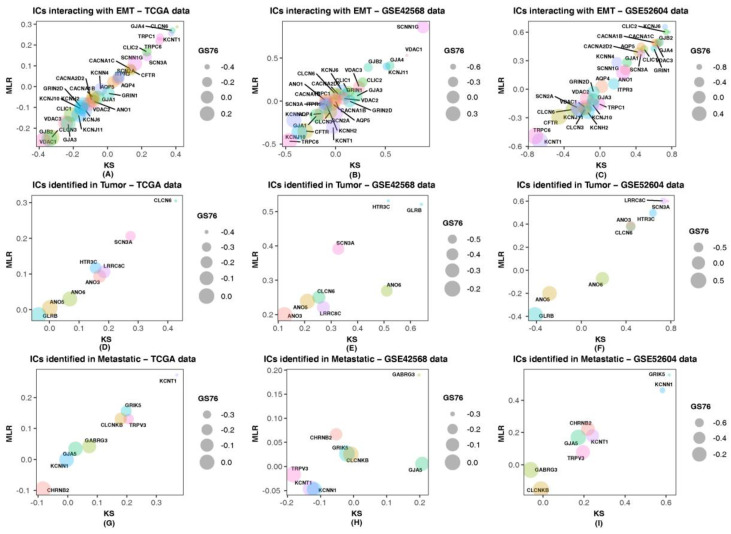
Correlation between ion channels (ICs) and EMT scores obtained using GS76, MLR and KS methods. In the bubble plot, the x-axis consists of correlation values of ion channels with the KS method, and the y-axis consists of correlation values with the MLR method. Each bubble corresponds to a particular ion channel represented by different colors. The size of the bubble corresponds to the correlation values of ion channels with GS76. TGCA data were used as RNA-Seq data and GSE42568 and GSE52604 were microarray datasets. (**A**–**C**) Correlation of ICs identified as interacting with EMT-related genes with GS76, MLR and KS scores; (**D**–**F**) Correlation of ICs identified in tumor state with GS76, MLR and KS scores; (**G**–**I**) Correlation of ICs identified in metastatic state with GS76, MLR and KS scores.

The ion channels *CLIC2*, *GJA4*, *HTR3C*, *CLCN6*, *SCN3A*, *ANO3*, *LRRC8C* and *GJA5* were found to be negatively correlated to GS76 and positively correlated to both KS and MLR scores in all three datasets, indicating that these ion channels might have a higher probability of contributing to acquiring and/or stabilizing a mesenchymal phenotype. Nevertheless, all ion channels other than *SCNN1G*, *CLCN3*, *KCNJ10* and *KCNH2* had a similar correlation trend in the expression values corresponding to one/more datasets. Figure 6 depicts the correlation of each of the identified ion channels with the EMT scores. The bubble plot represents the variation in the correlation of the genes with the three scoring methods. It landscapes the fact that the ion channels that may have a higher probability of contributing to the mesenchymal phenotype, correlating positively with the MLR and KS methods and negatively with the GS76 method.

### 3.5. Pathway Map of Identified Ion Channels and Their Associations with EMT

**Figure 7:**
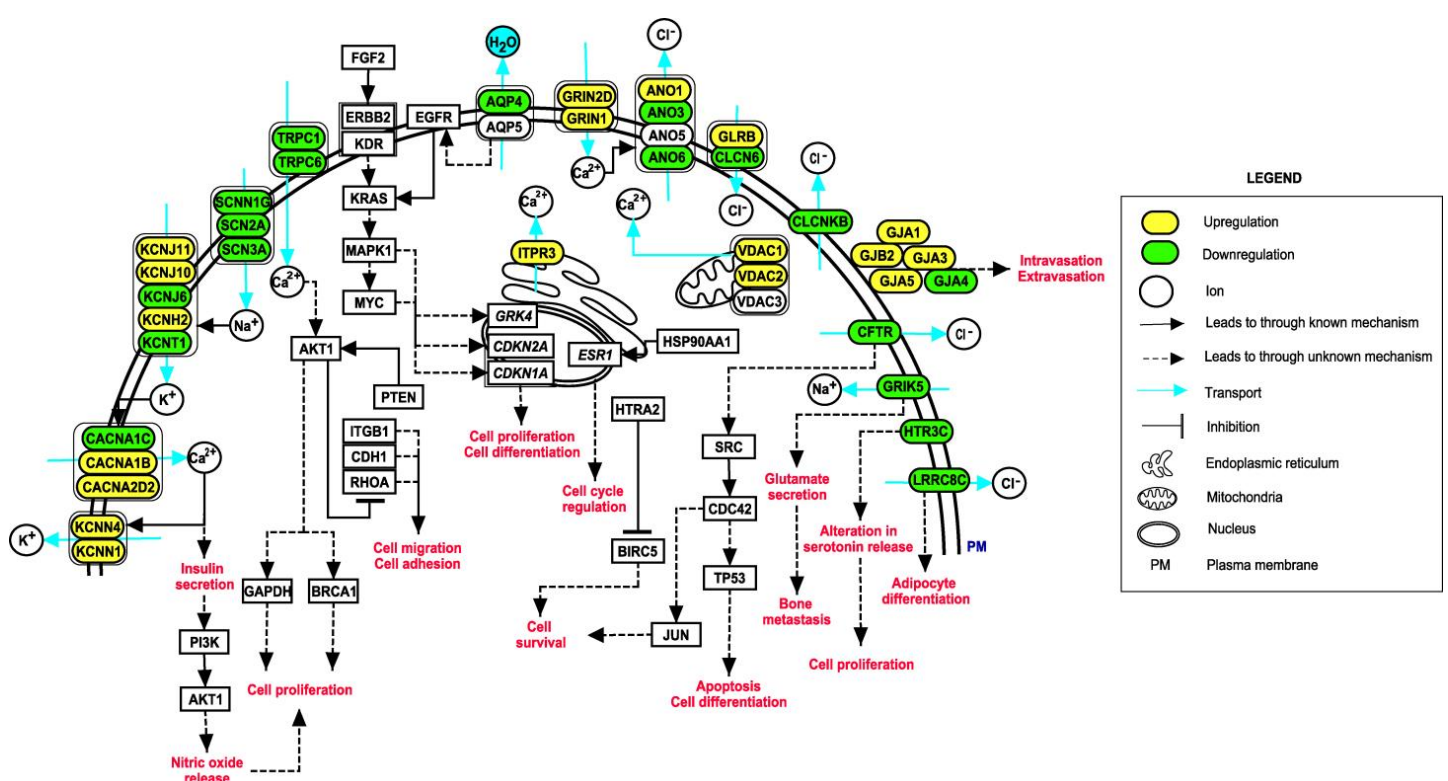
Depiction of events that may occur in breast cancer patients upon dysregulation of ion channels with EMT-related genes. The pathway map was generated using PathVisio (v3.3.0). Cancer phenotypes may appear due to alterations in important cellular processes, such as calcium signaling, insulin secretion, adipocyte metabolism, nitric oxide signaling and glutamatergic signaling.

Generation of pathway maps depicting potential ion channels in several cellular processes may aid in understanding their significance in various aspects of tumorigenesis in breast cancer patients. Figure 7 summarizes the overall identified ion channels and the events likely to occur in breast cancer patients when dysregulated ion channels function together with EMT-related genes. The pathway map consists of 66 molecules and 58 reactions. Several ion channels were differentially expressed and were found to have significant roles in key cellular processes.

### 3.6. Association of Identified Putative Ion Channels in Survival of Breast Cancer Patients

**Figure 8:**
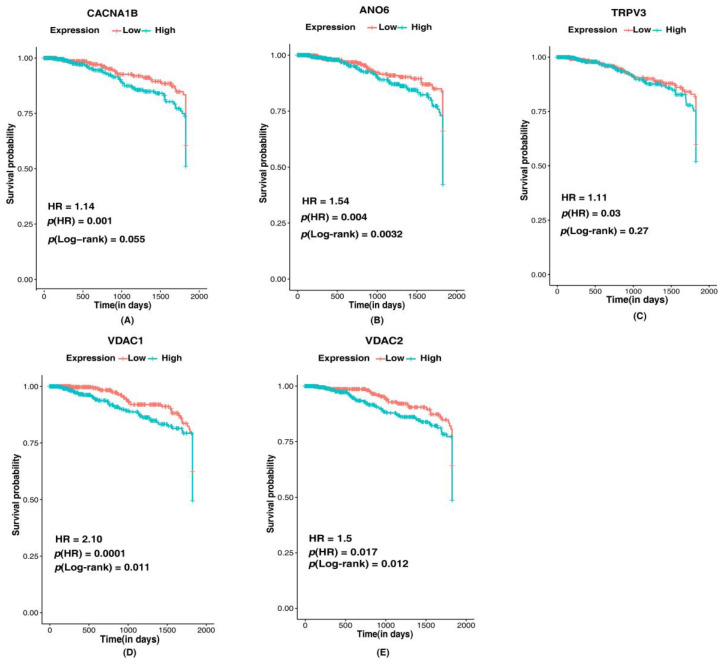
Kaplan–Meier 5-year survival curves representing the prognostic relationship between high and low expression of ion channels identified in breast cancer with survival probability. (**A**) *CACNA1B*, (**B**) *ANO6*, (**C**) *TRPV3*, (**D**) *VDAC1*, and (**E**) *VDAC2*.

The ion channels with HR > 1 and a *p*(HR) < 0.05 were selected as significant ion channels in survival of patients with breast cancer (Figure 8). Among the ion channels identified in association with EMT-related genes and tumor/ metastatic states of breast cancer, five ion channels—*CACNA1B* (HR: 1.14, *p*(HR): 0.001), *ANO6* (HR: 1.54, *p*(HR): 0.004), *TRPV3* (HR: 1.11, *p*(HR): 0.03), *VDAC1* (HR: 2.10, *p*(HR): 0.0001) and *VDAC2* (HR: 1.5, *p*(HR): 0.017) were found to be prognostically significant (Figure 8). The log-rank test *p*-value was used to evaluate the 5-year survival of the patients with altered expression of these ion channels. Patients with low expression of *CACNA1B* (*p*-value: 0.05), *ANO6* (*p*-value: 0.032), *TRPV3* (*p*-value: 0.27), *VDAC1* (*p*-value: 0.011) and *VDAC2* (*p*-value: 0.012) had better survival than patients with higher expression. Overall survival analysis of the ion channels using GEPIA in tumor state ion channels *ANO5* (*p*-value: 0.16)*, LRRC8C* (*p*-value: 0.48) and *GLRB* (*p*-value: 0.84) (Figure S5A–C) indicated high expression of genes correlated with poor survival. Expression of metastatic state ion channels *GRIK5* (*p*-value: 0.51) and *CLCNKB* (*p*-value: 0.18) (Figure S5D,E) were also associated with survival in breast cancer patients. Survival analysis of ion channels interacting with EMT-related genes showed *KCNN4* (*p*-value: 0.071)*, CFTR* (*p*-value: 0.16)*, KCNJ10* (*p*-value: 0.17)*, VDAC1* (*p*-value: 0.000003) and *VDAC2* (*p*-value: 0.052) (Figure S5F–J) the level of expression associated with survival of breast cancer patients. High expression of the ion channels (exception: *KCNN4*) could lead to poor patient survival. Low expression of *KCNN4* indicated poor survival of breast cancer patients.

## 4. Discussion

A deeper understanding of the role of ion channels in cells expressing cancerous phenotypes needs to be elucidated. Blockade of ion channels have been demonstrated to influence various pathophysiological conditions [40], making ion channels potential biomarkers in cancer diagnosis and therapeutics. This study focused on the identification of putative ion channels in tumor and metastatic states and their association with EMT programs of breast cancer through various computational analyses.

In the present study, analysis of transcriptomic data belonging to different platforms revealed the presence of several altered ion channels and EMT-related genes in patients with breast cancer. WGCNA of the altered ion channels led to the identification of significantly relevant co-expressed ion channels that were clustered into modules. Each of the selected modules consisted of a distinct set of ion channels. Although a few overlapping ion channels were noticed in the modules representing normal to tumor state and tumor to metastatic state of breast cancer. The normal to metastatic state modules appeared to be populated with a manifold of co-expressed ion channels as compared to the normal to tumor state modules. This could be an indication that as cancer progresses from normal to tumor and tumor to metastatic, the number of ion channels being deregulated aggravates.

Twenty-two unique ion channels in the tumor growth of breast cancer were identified through this study. Of those, *GJA1*, *AQP1*, *SCN4B* and *AQP7* were stated previously in breast cancer tumor growth [41,42,43,44]. Similarly, *PKD2*, *GJA4*, *KCNJ8*, *TRPC1*, *KCNJ2*, *KCND2*, *KCNB1*, *CLIC5*, *CLIC2* and *GABRE* were previously reported to have a role in tumor growth in several tumors [45,46,47,48,49,50,51,52,53,54,55,56]. *HTR3C*, *CLCN6*, *GLRB*, *SCN3A*, *ANO3*, *ANO5*, *ANO6* and *LRRC8C* were identified in this study as putative ion channels that may be implicated in tumor growth and development in breast cancer.

### 4.1. Ion Channels Identified as Putative Ion Channels in the Tumor State of Breast Cancer

- *HTR3C*, a ligand-gated ion channel, is one of the receptors for serotonin, a hormone that functions as a neurotransmitter and mitogen. Activation of this receptor causes fast, depolarizing responses. Additionally, *SCN3A*, a voltage gated sodium channel, is known to maintain depolarization in the enterochromaffin cells, which results in the regulation of serotonin release [57]. Serotonin functions as a tumor-suppressant in non-transformed breast cells and early-stage breast cancers. During tumor progression, cells acquire genetic or epigenetic alterations in serotonin signaling. This makes them resistant to suppressive actions of serotonin and favors tumor-promoting actions [58].
- *CLCN6* is a voltage-dependent chloride channel and has a role in regulating blood pressure levels and hypertension [59]. *cMyc* a protooncogene responsible for cell proliferation in various cancers, transcriptionally regulates GRK4 protein that was reported to be overexpressed in breast cancer tissues [60]. GRK4 has been demonstrated to be associated with an increased risk of hypertension, indicating hypertension as an important factor in breast cancer [60].
- *GLRB*, a glycine receptor, is a ligand-gated ion channel that mediates the inhibitory effects of glycine. Vascular endothelial growth factor (VEGF) has a crucial role in cancer progression as it promotes the formation of new blood vessels. Activation of VEGF receptor results in activation of phospholipase C-gamma and increases intracellular Ca^2+^ concentration. VEGF-induced cell proliferation is dependent on intracellular Ca^2+^ concentration. Hyperpolarization of the cell membrane due to glycine-gated chloride channels blocks the influx of Ca^2+^, thereby minimizing VEGF-mediated signaling. Thus, changes in the functioning of *GLRB* may promote tumor growth [61].
- Anoctamins (ANOs) are Ca^2+^ activated chloride channels. The activation of receptors of growth hormone signaling takes place through RAS-RAF-ERK, PI3K-AKT and DAG-IP3 pathways. It is known that anoctamin-controlled calcium channels are relevant for the activation of ERK-1,2. Additionally, the rise in intracellular Ca^2+^ together with activation of RAS-RAF/ERK pathway is a major aspect of cell proliferation [62]. *ANO1* has been widely studied in various tumors in this respect. However, very few studies have reported the involvement of other members of anoctamins. *ANO3, ANO5* and *ANO6* were identified in this study.
- *LRRC8C* is a volume-regulated anion channel (VRAC). VRACs are activated on cell swelling and play a critical role in cell volume regulation [63]. The *Fad158* gene, which has a crucial role in adipocyte differentiation, belongs to the LRRC8 family and is also known as *LRRC8C* [64]. As adipocytes are the major components of breast tissue, abnormal adipocyte metabolism leads to accumulation of tumor-supporting cells and other effects, such as insulin resistance, dyslipidemia and oxidative stress [65]. These effects may further lead to the aggressive nature of the tumor. Additionally, VRACs are known to mediate cellular uptake of drugs, such as cisplatin and carboplatin, which are widely used in the treatment of cancer [66]. However, LRRC8C was not reported to have a direct effect on the uptake of these drugs but was stated to have a possible role in mediating the action of LRRC8A [66].

The study also identified 22 unique ion channels in the metastatic state, of which *GRIK2*, *GJB3*, *KCNB2*, *KCNA1* and *KCNK2* were previously reported in breast cancer metastasis [67,68,69,70,71]. Similarly, *GABRG1*, *KCNQ3*, *GABRB2*, *GRIN2A*, *GRIA4*, *P2RX6*, *CACNA2D1*, *SCNN1G* and *TRPC4* were previously reported in metastasis in various other tumors [72,73,74,75,76,77,78,79,80,81,82,83]. *TRPV3*, *GABRG3*, *KCNT1*, *GJA5*, *KCNN1*, *GRIK5*, *CLCNKB* and *CHRNB2* were identified as putative ion channels that might play a crucial role in breast cancer metastasis.

### 4.2. Ion Channels Identified as Putative Ion Channels in the Metastatic State of Breast Cancer

- *TRPV3* is not a well-understood ion channel and can be activated by temperature. Although it has been reported as overexpressed in non-small cell lung cancer [84].
- *GABRG3* belongs to the GABA-A receptor gene family of hetero-pentameric ligand-gated ion channels. GABA-A receptor *GABRP* is required for maintaining basal-like cytokeratin expression, ERK1/2 phosphorylation and pro-migratory phenotype of breast cancer cells [85]. GABA-B receptors promote metastasis by enhancing ERK phosphorylation and thus activating metalloproteins that enable tumor cells to penetrate the basement membrane [86]. These studies indicate the importance of GABA receptors in the metastatic progression of breast cancer.
- *KCNT1* is a Na^+^ activated K^+^ channel with diverse functions, including insulin secretion and cell volume regulation. With the help of insulin receptors, insulin regulates endothelial cell migration, proliferation and production of VEGF. It also activates PI3K/Akt signaling that promotes nitric oxide (NO) release. NO increases endothelial survival, migration, proliferation and vascular permeability [87].
- *KCNN1* is a Ca^2+^ dependent potassium channel involved in regulating cell volume. Increased permeability of K^+^ due to activation of these channels results in membrane hyperpolarization. This enhances Ca^2+^ entry and thus contributes to a decrease in the regulatory volume of a cell [88].
- *CLCNKB* is a voltage-gated chloride channel. Chloride channels participate in cell volume regulation, membrane potential stabilization, signal transduction and transepithelial transport. *CLCNKB* has been widely studied in Bartter syndrome. Its role in breast cancer is not well established. These channels aid in maintaining intracellular ion concentration lower than extracellular ion concentration, preventing osmotic cell swelling. Altered expression in these channels may result in osmotic stress contributing to cell migration [89].
- *GJA5* is a member of the connexin family. Connexins are involved in the formation of heterologous gap junctions between tumor and endothelial cells, which facilitate intravasation and extravasation [90]. Various gap junctions have been reported in this regard. Very few studies have reported *GJA5* [91].
- *GRIK5* belongs to the glutamate-gated ionic family. Cells with breast cancer phenotype secrete high levels of glutamate and metastasize to bones. These cells with excess glutamate result in cancer-induced bone pain, which is a significant co-morbidity in advanced stage breast cancer patients [92].

A subset of ion channels, including GJA1, TRPC6, VDAC1 and AQP5, clustered together with hub EMT-related genes when PPI network analysis of the combined gene set was performed.

### 4.3. Ion Channels Interacting with Multiple Hub EMT-Related Genes in Breast Cancer

- GJA1 has roles in the cAMP pathway, Wnt signaling pathway and activation of p38. It was also reported as a tumor suppressor and a potent molecule in the acceleration of cancer progression [93].
- TRPC6 is present ubiquitously in human tissues and is involved in Ca^2+^ dependent pathways. It is also known to be upregulated in various other disease conditions [94].
- TRPC1, another ion channel involved in modulation of Ca^2+^, is an established biomarker in certain cancers [95].
- VDAC1 is a multifunctional channel involved in controlling the communications between mitochondria and the rest of the cell [96].
- ANO1 channel is involved in cell proliferation, survival, migration, contraction, secretion and neuronal excitation [97].
- AQP5, an aquaporin, is involved in the movement of water across cellular membranes. This process is one of the most important phenomena resulting in cell movement, cellular viscosity and signal transductions [39].
- CLIC1 was identified in maintenance of cell volume, ion homeostasis, trans-epithelial transport, pH regulation and cell cycle regulation [98].
- GJB2 regulates cell migration and colonization and thus aggressive phenotype in breast cancer [99].
- KCNH2 was reported to be involved in cell proliferation and cell migration processes implicating MAP kinase and c-fos pathways through a cell line study [100].
- CFTR, an anion channel, is known for its role in ion and acid-base homeostasis. It was also reported as a tumor suppressor [101].

An increasing number of studies are currently aiming to gain insights into the EMT program in cancer, which includes identifying and understanding the role of specific ion channels in the induction and maintenance of various aspects of EMT programs. The outcomes of these studies provide a preliminary perception that ion channels as therapeutic targets may be useful to control EMT in cancer. The involvement of putative ion channels in various cellular processes further substantiates its potential as a target. Thus, ion channels correlating with EMT-related genes identified through this study may aid in modulating several processes involved in tumor growth and progression and could act as promising targets. Kaplan–Meier plots and Cox regression analysis determined the prognostically significant ion channels in breast cancer among the putative ion channels. Overall, this study could identify various ion channels in breast cancer. Nevertheless, due to the data mining approach, the outcomes of the study may appear in the form of overfitting or underfitting. Thus, further theoretical and experimental studies need to be carried out to validate the obtained findings. Together, our analysis is a relevant data source for ion channels in breast cancer and may help in its management.

## 5. Conclusions

A systems biology-based approach was used to identify putative ion channels in tumor growth and metastasis and their correlation with EMT-related genes through analysis of RNA-Seq and microarray-based expression profiles of patients with breast cancer. Amid the identified ion channels, also present were ion channels already established as prognostic markers in breast cancer. Functional annotations of the altered ion channels led to the identification of processes contributing to cell proliferation, cell migration and cell volume regulation in breast cancer. However, detailed mechanisms underlying the possible effects of the identified ion channels and their associations with EMT need to be further characterized in vitro and in vivo.

## Figures and Tables

**Table 1:** A list of total upregulated and downregulated ion channels and EMT-related genes in HT, TM and HM in RNA-Seq (TCGA) and microarray (GSE42568, GSE52604) datasets.

Dataset	Upregulated			Downregulated		
	Ion Channels	EMT-Related Genes	Common Genes	Ion Channels	EMT-Related Genes	Common Genes
TCGA						
HT	145	343	6	80	264	-
TM	31	192	-	26	144	-
HM	71	311	3	70	290	-
GSE42568						
HT	12	150	1	16	176	-
TM	-	-	-	-	-	-
HM	13	181	2	16	163	-
GSE52604						
HT	-	-		-	-	-
TM	-	-		-	-	-
HM	35	310	2	120	160	5

**Table 2:** A partial list of differentially expressed ion channels in HT state in breast cancer.

Ion Channels	Description	Differential Expression
*HTR3C*	5-hydroxytryptamine receptor 3C	Downregulated
*CLCN6*	chloride voltage-gated channel 6	Downregulated
*GLRB*	glycine receptor beta	Upregulated
*SCN3A*	sodium voltage-gated channel alpha subunit 3	Downregulated
*ANO3*	anoctamin 3	Downregulated
*ANO6*	anoctamin 6	Downregulated
*LRRC8C*	leucine rich repeat containing 8 VRAC subunit C	Downregulated

**Table 3:** A partial list of differentially expressed ion channels in TM state in breast cancer.

Ion Channels	Description	Differential Expression
*TRPV3*	transient receptor potential cation channel subfamily V member 6	Downregulated
*GRIK5*	glutamate ionotropic receptor kainate type subunit 5	Downregulated
*CLCNKB*	chloride voltage-gated channel Kb	Downregulated
*GABRG3*	gamma-aminobutyric acid type A receptor gamma3 subunit	Upregulated
*KCNT1*	potassium sodium-activated channel subfamily T member 1	Downregulated
*KCNN1*	potassium calcium-activated channel subfamily N member 1	Downregulated
*GJA5*	gap junction protein alpha 5	Upregulated

**Table 4:** A list of ion channels interacting with the top 15 EMT-related proteins with the highest degree centrality measure in protein–protein interaction networks of modules on HT DEGs in breast cancer GSE42568 dataset.

Dataset	Degree	EMT-Related Proteins	Description	Interacting Ion Channels
HT-GSE42568	54	JUN	Jun proto-oncogene, AP-1 transcription factor subunit	TRPC6, GJA1
	54	MYC	MYC proto-oncogene, bHLH transcription factor	GJA1
	46	KRAS	KRAS proto-oncogene, GTPase	GRIN1, CLCN6
	44	FGF2	Fibroblast growth factor 2	GJA1
	44	PTEN	phosphatase and tensin homolog	GJA1
	43	KDR	kinase insert domain receptor	GJA1, SCN3A, SCN2A
	40	RHOA	ras homolog family member A	GJA1, TRPC6, TRPC1
	31	CAV1	caveolin 1	CACNA1B, GJA1, CLIC2, GJA4, TRPC1
	30	ITGB1	integrin subunit beta 1	GJA1
	29	CXCL12	C-X-C motif chemokine ligand 12	GJA1

**Table 5:** A list of ion channels interacting with the top 15 EMT-related proteins with the highest degree centrality measure in protein–protein interaction networks of modules on HT DEGs in breast cancer TCGA dataset.

Dataset	Degree	EMT-Related Proteins	Description	Interacting Ion Channels
HT-TCGA	74	GAPDH	glyceraldehyde-3-phosphate dehydrogenase	CLIC1, VDAC1, VDAC2, VDAC3
	46	SRC	SRC proto-oncogene, non-receptor tyrosine kinase	CLCN3, GJA3, KCNH2
	46	HSP90AA1	heat shock protein 90 alpha family class A member 1	KCNH2, VDAC1
	42	CDKN2A	cyclin dependent kinase inhibitor 2A	KCNJ11
	41	KRAS	KRAS proto-oncogene, GTPase	GRIN2D
	36	DNMT1	DNA methyltransferase 1	KCNJ10
	36	HSPA4	heat shock protein family A (Hsp70) member 4	KCNH2, VDAC1
	30	CDC42	cell division cycle 42	CACNA2D2

**Table 6:** A list of ion channels interacting with top 15 EMT-related proteins with the highest degree centrality measure in protein–protein interaction networks of modules on HM DEGs in breast cancer GSE42568 dataset.

Dataset	Degree	EMT-Related Proteins	Description	Interacting Ion Channels
HM-GSE42568	99	TP53	tumor protein p53	CFTR
	93	AKT1	AKT serine/threonine kinase 1	CLIC1
	90	GAPDH	glyceraldehyde-3-phosphate dehydrogenase	AQP4, AQP5, CACNA1C, CFTR, KCNN4, CLIC1
	90	CDH1	cadherin 1	ANO1, AQP5
	61	ERBB2	erb-b2 receptor tyrosine kinase 2	ANO1
	58	EZH2	enhancer of zeste 2 polycomb repressive complex 2 subunit	CACNA1C
	47	BRCA1	BRCA1, DNA repair associated	CFTR
	42	DNMT1	DNA methyltransferase 1	CFTR

**Table 7:** A list of ion channels interacting with top 15 EMT-related proteins with the highest degree centrality measure in protein–protein interaction networks of modules on HM DEGs in breast cancer GSE52604 dataset.

Dataset	Degree	EMT-Related Proteins	Description	Interacting Ion Channels
HM-GSE52604	117	AKT1	AKT serine/threonine kinase 1	ITPR3, CLIC1, GJB2
	116	CDH1	cadherin 1	GJB2
	84	NOTCH1	notch 1	KCNT1
	75	MAPK1	mitogen-activated protein kinase 1	SCNN1G

**Table 8:** A list of ion channels interacting with top 15 EMT-related proteins with the highest degree centrality measure in protein–protein interaction networks of modules on HM DEGs in breast cancer TCGA dataset.

Dataset	Degree	EMT-Related Proteins	Description	Interacting Ion Channels
HM-TCGA	59	GAPDH	glyceraldehyde-3-phosphate dehydrogenase	CLIC1, VDAC1, VDAC3
	41	HSP90AA1	heat shock protein 90 alpha family class A member 1	KCNH2, VDAC1
	34	CDKN2A	cyclin dependent kinase inhibitor 2A	KCNJ11
	33	DNMT1	DNA methyltransferase 1	KCNJ10
	31	HSPA4	heat shock protein family A (Hsp70) member 4	KCNH2, VDAC1
	25	BIRC5	baculoviral IAP repeat containing 5	KCNJ6
	24	CDC42	cell division cycle 42	CACNA2D2

**Table 9:** Correlation between GS76, MLR and KS EMT scoring methods across samples.

Dataset	GS76.MLR-Cor	GS76.MLR-*p*val	GS76.KS-Cor	GS76.KS-*p*val	KS.MLR-Cor	KS.MLR-*p*val
TCGA	−0.843467533	<0.00001	−0.803881144	0.00	0.718122546	6.1098 × 10^−221^
GSE42568	−0.863655242	<0.00001	−0.876536316	<0.00001	0.770347822	<0.00001
GSE52604	−0.677929378	<0.00001	−0.923135739	<0.00001	0.673478229	<0.00001

## Data Availability

Publicly available datasets were analyzed in this study. The data can be accessed using the following resources: UCSC Xena portal (https://xena.ucsc.edu/) accessed on 25 March 2021, TGCA GDC data portal (https://portal.gdc.cancer.gov/) accessed on 25 March 2021, GTEx portal (https://gtexporatal.org/home/datasets/) accessed on 25 March 2021, GEO database (https://www.ncbi.nlm.nih.gov/geo/) accessed on 25 March 2021. Customized R scripts used for identification of DEGs and co-expressed gene modules and the breast cancer ICs-EMT pathway reaction data are available in the GPML format through the GitHub repository via the following URLs: (https://github.com/js-iob/BreastCancer_ICs-EMT/blob/main/BreastCancer_ICs-EMT_PathwayMap.gpml, https://github.com/js-iob/BreastCancer_ICs-EMT/blob/main/WGCNA.R, https://github.com/js-iob/BreastCancer_ICs-EMT/blob/main/differential_expression_Microarray.R, https://github.com/js-iob/BreastCancer_ICs-EMT/blob/main/differential_expression_RNASeq.R, https://github.com/js-iob/BreastCancer_ICs-EMT/blob/main/survival.R) accessed on 26 January 2022.

## Supplementary Materials

The following are available online at https://www.mdpi.com/article/10.3390/cancers14061444/s1, Table S1: Clinical Information of breast cancer patients from TCGA and GTEx datasets, Table S2: Clinical information of breast cancer patients in GSE52604 dataset, Table S3: Clinical information of breast cancer patients in GSE42568 dataset, Table S4: non-redundant list of differentially expressed ion channels in HM, NT and TM corresponding to patients with breast cancer, Table S5: non-redundant list of differentially expressed EMT-related genes in HM, NT and TM corresponding to patients with breast cancer, Table S6: Differential expression of ion channels overlapped in EMT gene-set, Table S7: Parameters used for WGCNA analysis of ion channel gene-set in HT, TM and HM, Table S8: Parameters used for WGCNA analysis of ion channels integrated with EMT gene-set in HT, TM and HM, Table S9: A list of samples present in RNA-Seq dataset and their corresponding EMT score estimated using GS76, MLR and KS EMT scoring methods, Table S10: A list of samples present in GSE42568 dataset and their corresponding EMT score estimated using GS76, MLR and KS EMT scoring methods, Table S11: List of samples present in GSE52604 dataset and their corresponding EMT score estimated using GS76, MLR and KS EMT scoring methods, Table S12: Correlation of expression values of ion channels identified as interacting with EMT and ICs identified in tumor and metastatic states belonging to RNA-Seq, GSE42568 and GSE52604 with GS76, MLR and KS EMT scoring methods, Figure S1: Co-expressed ion channel modules based on the non-redundant ion channel DEGs: (1) Identification of gene modules in HM based on the list of non-redundant HM DEGs. (2) Identification of gene modules in TM based on the list of non-redundant TM DEGs. (A) Gene module identification of expression data in RNA-Seq dataset. (B) Gene module identification of expression data in GSE42568 dataset (i) Determination of soft-thresholding power for WGCNA analysis (ii) Hierarchical clustering of genes into modules. Modules are assigned different colours as depicted in the horizontal bar below the tree diagram. (iii) Module-trait relationship plot for normal, tumor and metastatic samples. Rows correspond to modules depicted as different colours and columns are the binary traits. Numbers in each cell are the correlation coefficient between module eigengenes and the binary traits and the corresponding p-value (iv) Scatter plot of gene significance for the binary trait vs the module membership in selected module. Figure S2: Co-expressed ion channel modules based on the non-redundant ion channel and EMT DEGs: (1) Identification of gene modules in HM based on the list of non-redundant HM DEGs. (2) Identification of gene modules in TM based on the list of non-redundant TM DEGs. (3) Identification of gene modules in HT based on the list of non-redundant HT DEGs. (A) Gene module identification of expression data in RNA-Seq dataset. (B) Gene module identification of expression data in GSE42568 dataset. (C) Gene module identification of expression data in GSE52604 dataset. (i) Determination of soft-thresholding power for WGCNA analysis (ii) Hierarchical clustering of genes into modules. Modules are assigned different colours as depicted in the horizontal bar below the tree diagram. (iii) Module-trait relationship plot for normal, tumor and metastatic samples. Rows correspond to modules depicted as different colours and columns are the binary traits. Numbers in each cell are the correlation coefficient between module eigengenes and the binary traits and the corresponding p-value (iv) Scatter plot of gene significance for the binary trait vs the module membership in selected module. Figure S3: Representation of co-expression networks (CN) of selected modules containing combined gene-set. Black nodes represent EMT-related genes and red nodes represent ion channels. The edges represent the weights corresponding to each interaction. Higher the weight obtained through WGCNA darker is the edge (A) CN for microarray (GSE42568) data based on the list of non-redundant HT DEGs. (B) CN for microarray (GSE42568) data based on the list of non-redundant HM DEGs. (C) CN for microarray (GSE42568) data based on the list of non-redundant TM DEGs. (D) CN for microarray (GSE52604) data based on the list of HM DEGs (E) CN for RNA-Seq (TCGA) data based on the list of non-redundant HT DEGs. (F) CN for RNA-Seq (TCGA) data based on the list of non-redundant HM DEGs. (G) CN for RNA-Seq (TCGA) based on the list of non-redundant TM DEGs. Figure S4: Representation of protein-protein interaction networks (PPIN) of combined gene-set. Nodes in shades of blue represent EMT genes and yellow nodes represent ion channels. (A) PPIN for microarray (GSE42568) based on the list of non-redundant TM DEGs (B) PPIN for microarray (GSE52604) based on the list of non-redundant HM DEGs. (C) PPIN for RNA-Seq data based on the list of non-redundant HTDEGs. (D) PPIN for RNA-Seq data based on the list of non-redundant HM DEGs. (E) PPIN for RNA-Seq data based on the list of non-redundant TM DEGs. Figure S5: Kaplan-Meier survival curves representing the prognostic relationship between high and low expression of ion channels identified in breast cancer to overall survival (A) ANO5, (B) LRRC8C, (C) GLRB, (D) GRIK5 (E) CLCNKB, (F) CFTR, (G) KCNJ10, (H) KCNN4, (I) VDAC1, (J) VDAC2.
