# Supplementary material for: In Silico Analysis of Ion Channels and Their Correlation with Epithelial to Mesenchymal Transition in Breast Cancer
Source: Cancers (Basel). 2022 Mar 11;14(6):1444. doi: 10.3390/cancers14061444 (PMC8946083; doi:10.3390/cancers14061444)
Supplement: Supplementary file 1 [file cancers-14-01444-s001.zip › Supplementary_data.pdf]

Supplementary Data

Table S1: Clinical Information of breast cancer patients from TCGA and GTEx datasets

	**Total number of samples**
**Number of Individuals**	1389
**Average Age (Years)**	58
**Histology Type**	
Tumor	1090
Metastatic	7
Normal (TCGA)	113
Normal (GTEx)	179
**Disease Type**	
Ductal and Lobular neoplasms	1168
Complex Epithelial neoplasms	15
Adenomas and Adenocarcinomas	3
Epithelial Neoplasms	5
Cystic, Mucinous and Serous Neoplasms	16
Fibroepithelial Neoplasms	1
Squamous Cell Neoplasms	2

Table S2: Clinical information of breast cancer patients in GSE52604 dataset

	**Total number of samples**
**Number of Individuals**	55
**Histology Type**	
Metastatic	35
Normal (Brain)	10
Normal (Breast)	10

Table S3: Clinical information of breast cancer patients in GSE42568 dataset

	**Total number of samples**
**Number of Individuals**	121
**Average Age (Years)**	58
**Average Tumor Size (cm)**	2.79
**Histology Type**	
Tumor	45
Metastatic	59
Normal	17
**Pathological Tumor Stage**	
T1	18
T2	83
T3	3
**Pathological Grade**	
Grade1	11
Grade2	40
Grade3	53
**ER Status**	
ER^{+ve}	67
ER^{-ve}	34
Unknown	3
**Disease Type**	
Invasive ductal carcinoma	82
Invasive lobular	17
Tubular	2
Mucinous	3

Table S4: non-redundant list of differentially expressed ion channels in HM, NT and TM corresponding to patients with breast cancer (Excel sheet)

Table S5: non-redundant list of differentially expressed EMT-related genes in HM, NT and TM corresponding to patients with breast cancer (Excel sheet)

Table S6: Differential expression of ion channels overlapped in EMT gene-set

**Gene Symbol**	**GSE42568**				**GSE52604**		**RNA-Seq**					
	HM		HT		HM		HM		HT		TM	
	EMT	IC	EMT	IC	EMT	IC	EMT	IC	EMT	IC	EMT	IC
AQP3	-	-	-	-	-	-	↑	↑	↑	↑	-	-
AQP5	-	-	-	-	↑	↑	↑	↓	↑	↑	↓	↑
AQP9	-	-	-	-	↓	↓	-	↑	-	↑	-	-
CFTR	-	-	-	-	↓	↓	↓	-	-	-	-	-
GJB1	-	-	-	-	↓	↓	-	-	↑	↑		
GJB2	↑	↑	↑	↑	-	-	↑	↑	↑	↑	↓	↑
GRIN1	-	-	-	-	-	-	-	-	-	↑	-	-
KCNN4	↑	↑	-	-	↑	↑	↑	-	-	-	↓	-
KCNH1	-	-	-	-	↓	↓	↑	↑	↑	↑	-	-
TRPC5	-	-	-	-	↓	↓	-	-	-	-	-	-
TRPM8	-	-	-	-	-	-	-	↑	↑	↑	↑	-

Table S7: Parameters used for WGCNA analysis of ion channels gene-set in HT, TM and HM

Dataset	Subgroup	Good sample genes	Soft threshold power	Minimum module size	Deep split	Obtained number of modules	Chosen module
RNA-Seq	HT	1389:226	7	20	3	4	Turquoise
	TM	1389:57	6	10	4	2	Turquoise
	HM	1389:220	6	20	2	2	Turquoise
GSE42568	HT	121:221	10	20	4	4	Yellow
	TM	121:57	6	10	1	2	Turquoise
	HM	121:217	9	20	4	4	Brown
GSE52604	TM	55:55	7	10	0	1	Turquoise
	HM	55:219	10	20	2	2	Turquoise

Table S8: Parameters used for WGCNA analysis of ion channels integrated with EMT gene-set in HT, TM and HM

Dataset	Subgroup	Good sample genes	Soft threshol d power	Minimu m module size	Deep split	Obtaine d number of modules	Chosen module
RNA-Seq	HT	1389:926	12	30	3	6	Blue
	TM	1389:391	12	30	3	4	Blue
	HM	1389:1018	12	30	3	7	Blue
GSE42568	HT	121:913	6	30	1	6	Turquoise
	TM	121:385	8	30	1	3	Blue
	HM	121:1010	6	30	1	6	Blue
GSE52604	TM	55:380	12	30	3	3	Brown
	HM	55:998	12	30	3	6	Blue

Table S9: A list of samples present in RNA-Seq dataset and their corresponding EMT score estimated using GS76, MLR and KS EMT scoring methods (Excel sheet)

Table S10: A list of samples present in GSE42568 dataset and their corresponding EMT score estimated using GS76, MLR and KS EMT scoring methods (Excel sheet)

Table S11: List of samples present in GSE52604 dataset and their corresponding EMT score estimated using GS76, MLR and KS EMT scoring methods (Excel sheet)

Table S12: Correlation of expression values of ion channels identified as interacting with EMT and ion channels identified in tumor and metastatic states belonging to RNA-Seq, GSE42568 and GSE52604 with GS76, MLR and KS EMT scoring methods (Excel sheet)

Supplementary Figures

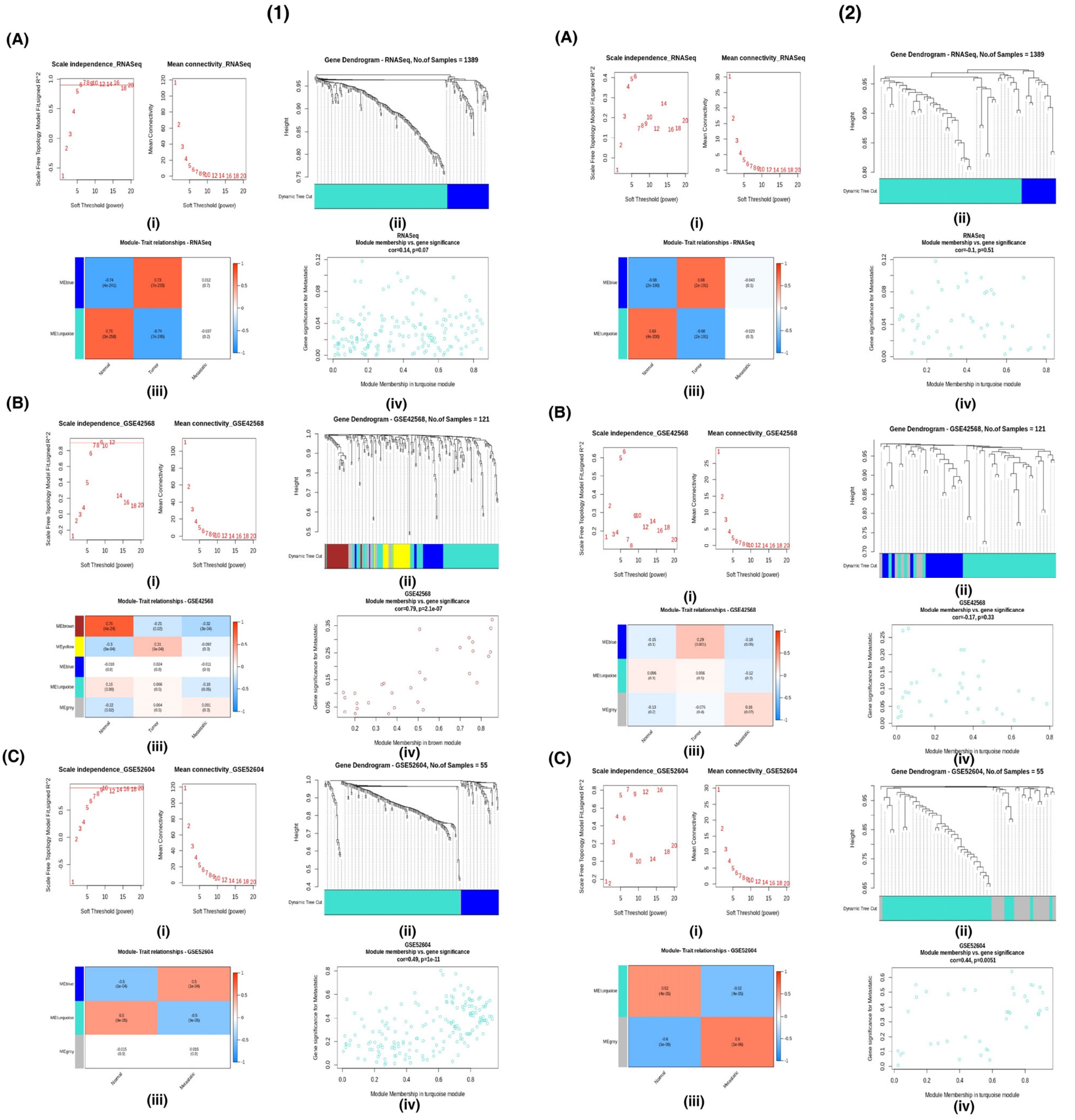

Figure S1: Co-expressed ion channel modules based on the non-redundant ion channel DEGs: (1) Identification of gene modules in HM based on the list of non-redundant HM DEGs. (2) Identification of gene modules in TM based on the list of non-redundant TM DEGs. (A) Gene module identification of expression data in RNA-Seq dataset. (B) Gene module identification of expression data in GSE42568 dataset (i) Determination of soft-thresholding power for WGCNA analysis (ii) Hierarchical clustering of genes into modules. Modules are assigned different colours as depicted in the horizontal bar below the tree diagram. (iii) Module-trait relationship plot for normal, tumor and metastatic samples. Rows correspond to modules depicted as different colours and columns are the binary traits. Numbers in each cell are the correlation coefficient between module eigengenes and the binary traits and the corresponding p-value (iv) Scatter plot of gene significance for the binary trait vs the module membership in selected module.

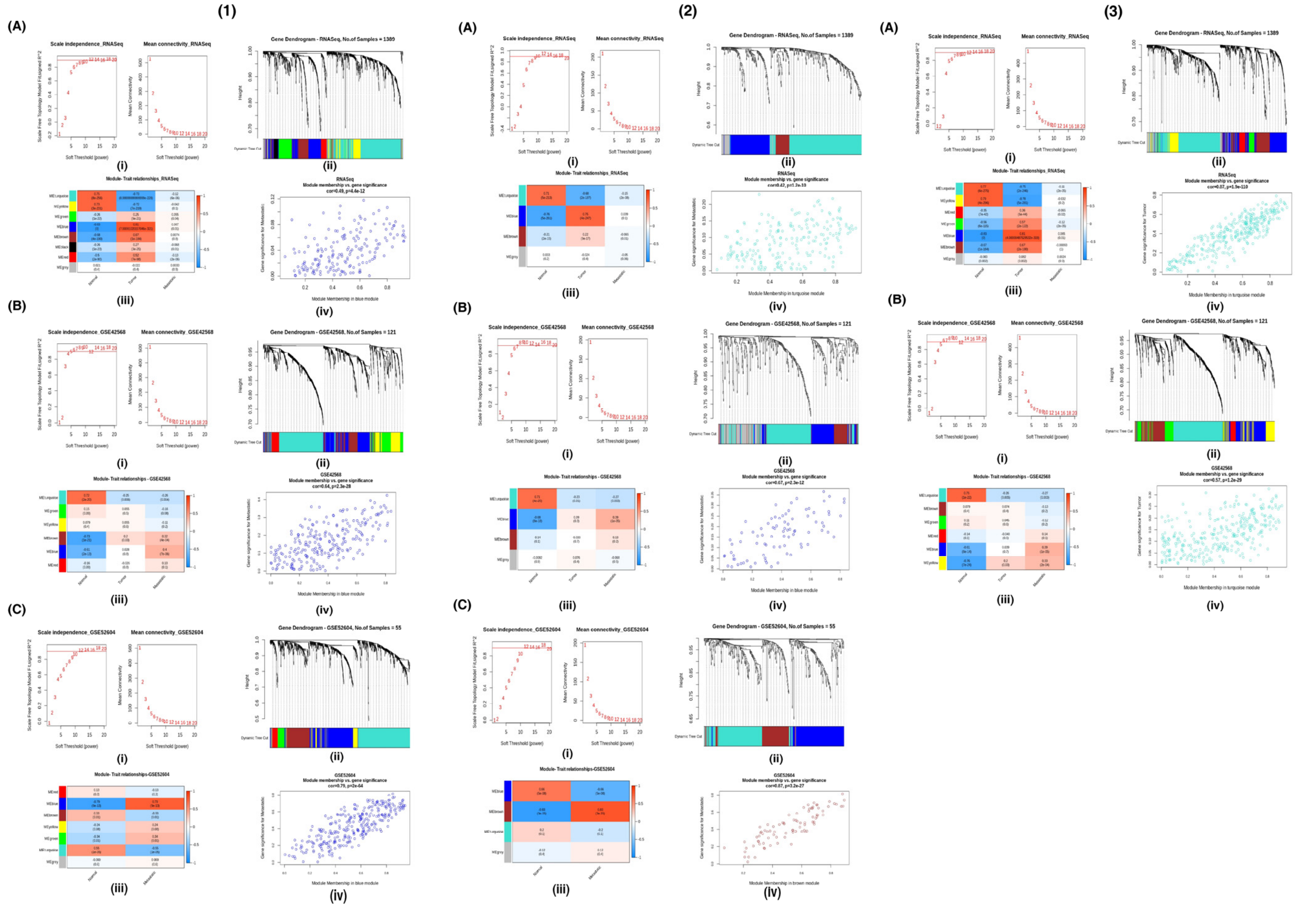

Figure S2: Co-expressed ion channel modules based on the non-redundant ion channel and EMT DEGs: (1) Identification of gene modules in HM based on the list of non-redundant HM DEGs. (2) Identification of gene modules in TM based on the list of non-redundant TM DEGs. (3) Identification of gene modules in HT based on the list of non-redundant HT DEGs. (A) Gene module identification of expression data in RNA-Seq dataset. (B) Gene module identification of expression data in GSE42568 dataset. (C) Gene module identification of expression data in GSE52604 dataset. (i) Determination of soft-thresholding power for WGCNA analysis (ii) Hierarchical clustering of genes into modules. Modules are assigned different colours as depicted in the horizontal bar below the tree diagram. (iii) Module-trait relationship plot for normal, tumor and metastatic samples. Rows correspond to modules depicted as different colours and columns are the binary traits. Numbers in each cell are the correlation coefficient between module eigengenes and the binary traits and the corresponding p-value (iv) Scatter plot of gene significance for the binary trait vs the module membership in selected module.

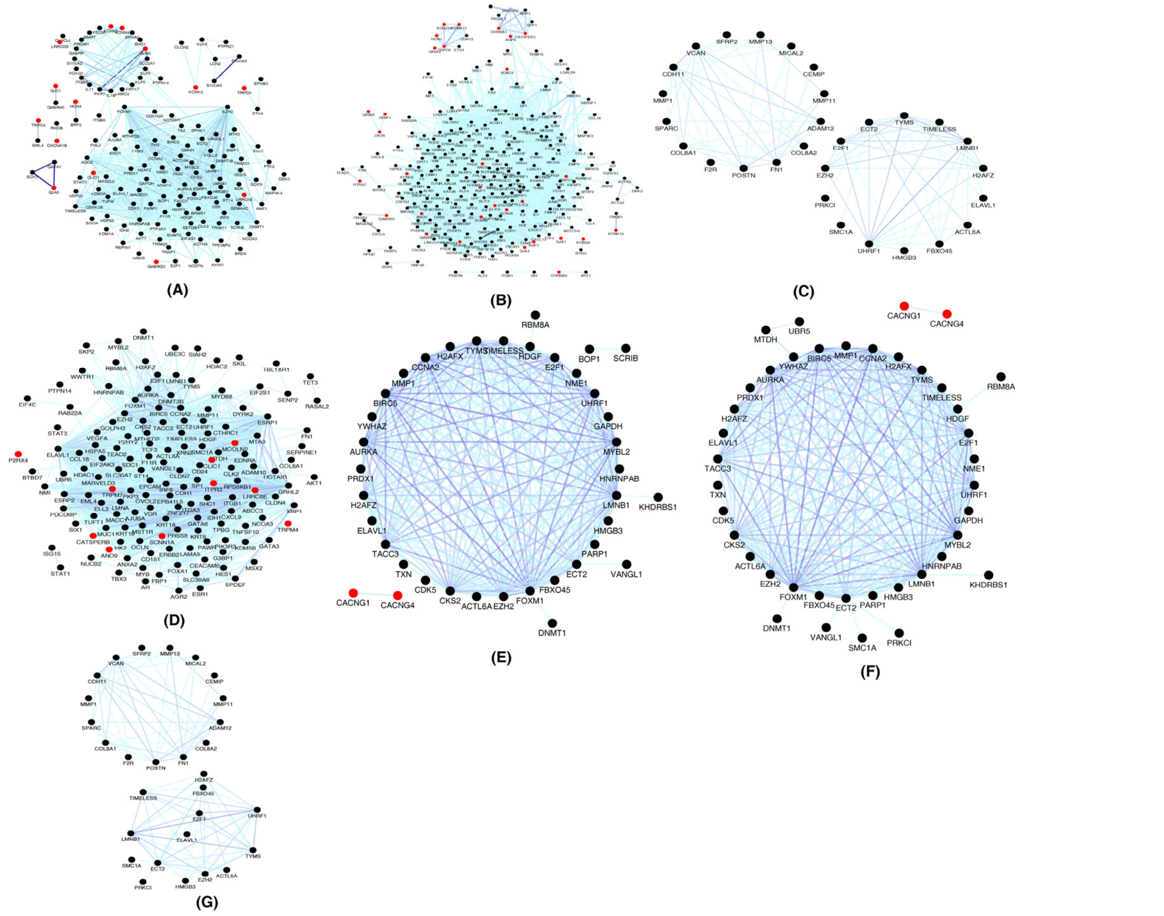

Figure S3: Representation of co-expression networks (CN) of selected modules containing combined gene-set. Black nodes represent EMT-related genes and red nodes represent ion channels. The edges represent the weights corresponding to each interaction. Higher the weight obtained through WGCNA darker is the edge (A) CN for microarray (GSE42568) data based on the list of non-redundant HT DEGs. (B) CN for microarray (GSE42568) data based on the list of non-redundant HM DEGs. (C) CN for microarray (GSE42568) data based on the list of non-redundant TM DEGs. (D) CN for microarray (GSE52604) data based on the list of HM DEGs (E) CN for RNA-Seq (TCGA) data based on the list of non-redundant HT DEGs. (F) CN for RNA-Seq (TCGA) data based on the list of non-redundant HM DEGs. (G) CN for RNA-Seq (TCGA) based on the list of non-redundant TM DEGs.

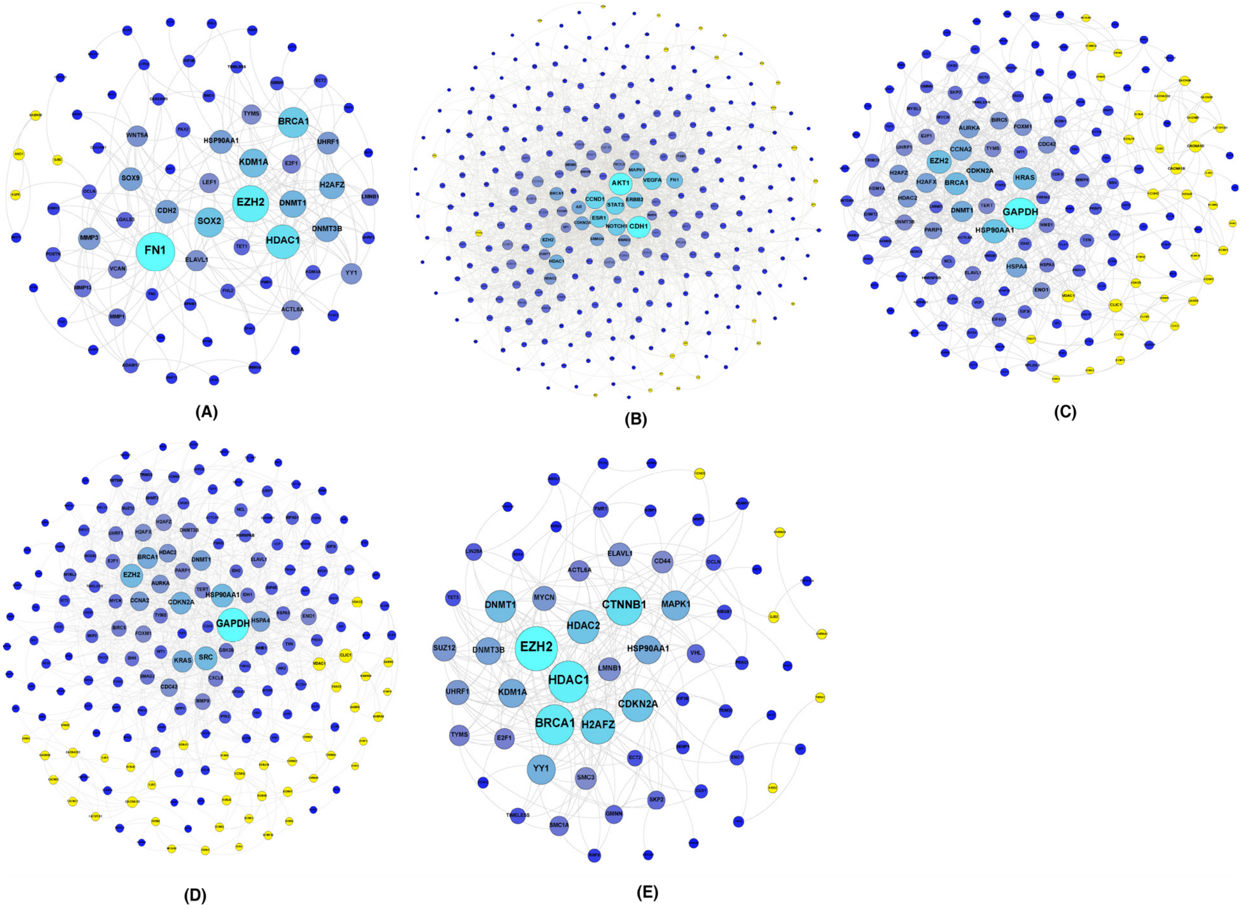

Figure S4: Representation of protein-protein interaction networks (PPIN) of combined gene-set. Nodes in shades of blue represent EMT genes and yellow nodes represent ion channels. (A) PPIN for microarray (GSE42568) based on the list of non-redundant TM DEGs (B) PPIN for microarray (GSE52604) based on the list of non-redundant HM DEGs. (C) PPIN for RNA-Seq data based on the list of non-redundant HTDEGs. (D) PPIN for RNA-Seq data based on the list of non-redundant HM DEGs. (E) PPIN for RNA-Seq data based on the list of non-redundant TM DEGs.

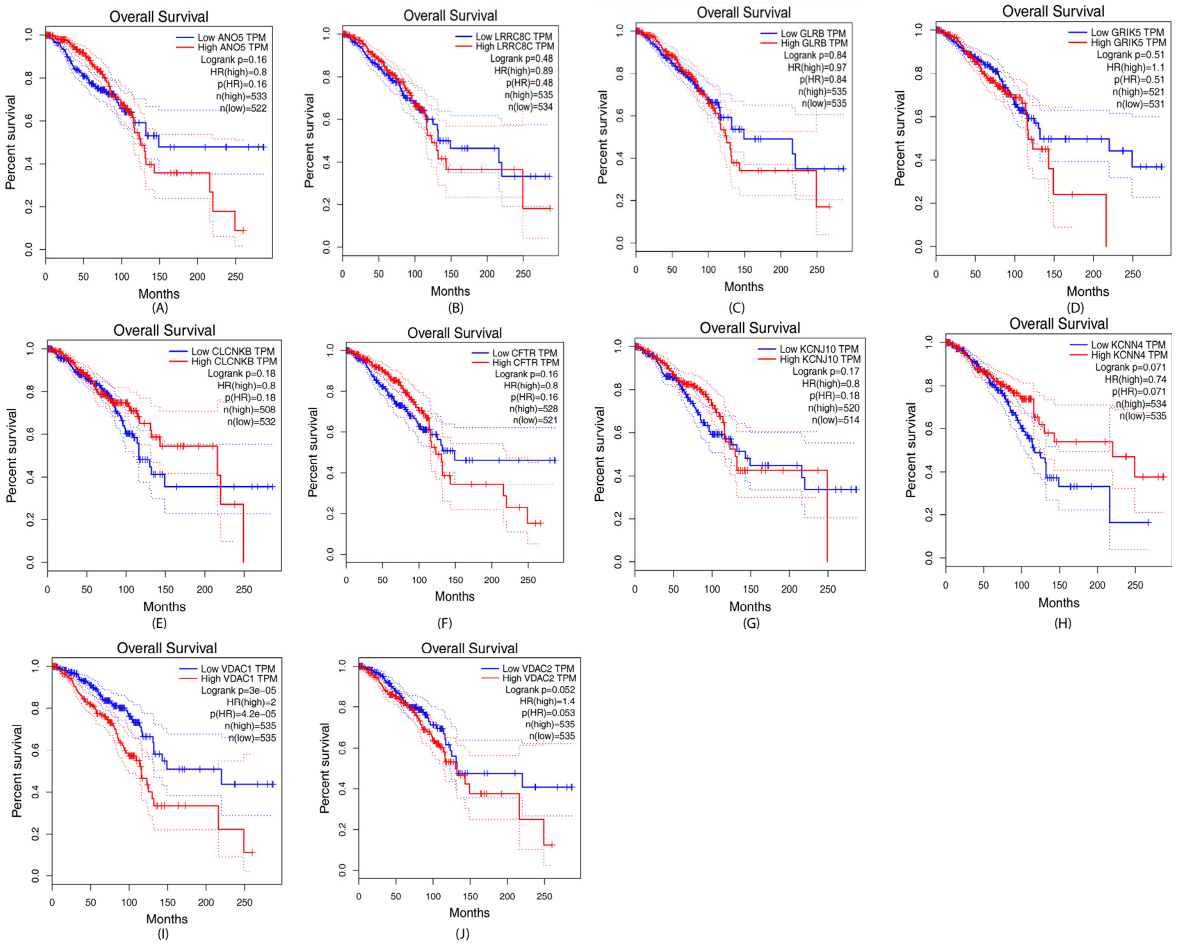

Figure S5: Kaplan-Meier survival curves representing the prognostic relationship between high and low expression of ion channels identified in breast cancer to overall survival (A) *ANO5*, (B) *LRRC8C*, (C) *GLRB*, (D) *GRIK5* (E) *CLCNKB*, (F) *CFTR*, (G) *KCNJ10*, (H) *KCNN4*, (I) *VDAC1*, (J) *VDAC2*
